# Activation of AKT negatively regulates the pro-apoptotic function of death-associated protein kinase 3 (DAPK3) in prostate cancer

**DOI:** 10.1016/j.canlet.2016.04.028

**Published:** 2016-07-28

**Authors:** Trinath P. Das, Suman Suman, A.M. Sashi Papu John, Deeksha Pal, Angelena Edwards, Houda Alatassi, Murali K. Ankem, Chendil Damodaran

**Affiliations:** a Department of Urology, University of Louisville, Louisville, KY, USA; b Department of Pathology, University of Louisville, Louisville, KY, USA

**Keywords:** Prostate cancer, Progression, Tumor suppressor, Oncogene, Molecular signaling

## Abstract

The activation of AKT governs many signaling pathways and promotes cell growth and inhibits apoptosis in human malignancies including prostate cancer (CaP). Here, we investigated the molecular association between AKT activation and the function of death-associated protein kinase 3 (DAPK3) in CaP. An inverse correlation of pAKT and DAPK3 expression was seen in a panel of CaP cell lines. Inhibition of AKT by wortmannin/LY294002 or overexpression of DAPK3 reverts the proliferative function of AKT in CaP cells. On the other hand, ectopic expression of AKT inhibited DAPK3 function and induced proliferation of CaP cells. In addition, AKT over-expressed tumors exhibit aggressive growth when compared to control vector in xenograft models. The immunohistochemistry results revealed a down-regulation of DAPK3 expression in AKT over-expressed tumors as compared to control tumors. Finally, we examined the expression pattern of AKT and DAPK3 in human CaP specimens – the expected gradual increase and nuclear localization of pAKT was seen in higher Gleason score samples versus benign hyperplasia (BPH). On the contrary, reduced expression of DAPK3 was seen in higher Gleason stages versus BPH. This suggests that inhibition of DAPK3 may be a contributing factor to the carcinogenesis of the prostate. Understanding the mechanism by which AKT negatively regulates DAPK3 function may suggest whether DAPK3 can be a therapeutic target for CaP.

## Highlights

• This is the first study that demonstrates the inverse correlation of AKT activation and down-regulation of tumor suppressor protein, DAPK-3, in CaP cell lines as well as human prostate tumor tissues that correlate with disease progression.
• Either silencing AKT or overexpressing DAPK-3 induces apoptosis in Castration Resistant Prostate Cancer cells.

## Introduction

Carcinoma of prostate (CaP) is the most common malignancy in men worldwide and especially in the United States [1]. Although several factors contribute to the etiology of prostate carcinogenesis, deletion or mutation of tumor suppressor genes facilitates the oncogenes activation. This plays a significant role in many cancer types [2], [3]. Deletion of the tumor suppressor phosphatase and tensin homolog (*PTEN)* induces PI3K/AKT-mediated oncogenic signaling and appears to be a critical event for human CaP [4], [5]. Recent studies have demonstrated that AKT phosphorylation (Ser^473^) leads to progression of castration-resistant prostate cancer (CRPC) and is correlated with poor clinical outcome [6]. Activated AKT not only promotes cell survival, proliferation, invasion and migration but also inhibits apoptosis by suppressing tumor suppressor genes like Fork head transcription factor class O3a (FOXO3a) [7], [8], prostate apoptosis response-4 (Par-4) [9], and BAD [10], [11].

Death-associated protein kinase (DAP-kinase) was recently identified and involves a wide array of apoptotic functions, which are regulated by many pro-apoptotic genes such as interferon (IFN)-γ, tumor necrosis factor (TNF)-α and Fas [12], [13]. The DAPK3 executes the pro-apoptotic function either by inducing apoptosis or activating autophagy with or without involvement of caspases [14], [15]. Phosphorylation of myosin light chain (MLC), a substrate of DAPK, causes membrane blebbing and induction of autophagy-mediated cell death in CaP [16], [17], [18]. It has been reported that DAPK3 is frequently methylated [19] or mutated [19] in many cancer types. This results in a loss of tumor suppression via DAPK3 in cancer.

Here, we demonstrate the inverse correlation of AKT activation and down-regulation of DAPK-3 in CaP cell lines as well as human prostate tumor tissues that correlate with disease progression. Either silencing AKT or overexpressing DAPK-3 induces apoptosis in CRPC cells. These studies suggest that activated AKT may down-regulate the pro-apoptotic function of DAPK-3; hence, either ectopic expression of DAPK3 or activation by small molecules may inhibit the progression of CaP.

## Materials and methods

### Cell lines, antibodies, and reagents

Human prostate carcinoma cell lines (PC-3 DU-145, CWR22RV1 and LNCaP) were obtained from American Type Cell Culture (ATCC, Manassas, VA) and cultured according to the guidelines of ATCC. The following antibodies were obtained from Cell Signaling Technology (Danvers, MA) and were used for the immunoblotting: anti-AKT, anti-pAKT, anti-DAPK3, anti-cleaved caspase-9, anti-cleaved caspase-3 and anti-cleaved PARP. Anti-mouse, anti-goat, and anti-rabbit secondary antibodies conjugated with HRP were purchased from Santa Cruz Biotechnology (Santa Cruz, CA). Annexin-FITC kit was purchased from BD Biosciences (San Diego, CA). Propidium iodide was purchased from Sigma (St. Louis, MO). Alexa Fluor 488, phalloidin, and prolong gold antifade with DAPI mountant were purchased from Invitrogen (Grand Island, NY). Mammalian expression plasmids for DAPK3 and control vectors were obtained from Origene (Cambridge, MA).

### Cell proliferation assays

Cells were treated with wortmannin (0.5-1 µM), LY294002 (25 µM) or DMSO (Vehicle) for 24 h. To confirm the viability of cells, MTT assay was performed following the manufacturer's protocol [20], [21], [22], [23].

### Protein extraction and western blotting

For western blotting whole cell lysates were prepared with Mammalian Protein Extraction Reagent (Thermo Scientific) according to the manufacturer's protocol. Western blotting was performed using specific antibodies against DAPK3, Actin, GAPDH, AKT, pAKT (Ser473 and Thr308), cleaved caspase-9, cleaved caspase-3 and cleaved PARP; expression was detected by chemiluminescence [20], [21], [22], [23].

### Overexpression of DAPK3

CaP cells in exponential growth phase were plated 12–16 h before transfection at a density of 5 × 10^5^ cells/well in six-well plates. Cells were transfected with either pCMV backbone vector or pCMV-DAPK3 expression plasmid using a lipofectamine transfection reagent (ThermoFisher Scientific, Waltham, MA) according to the manufacturer's protocol.

### Immunofluorescence staining

PC-3 cells (5 × 10^5^) were seeded in suspension onto coverslips in a six-well plate. After 24 h, the cells were transfected with 1 or 2 µg DAPK3 overexpression plasmid for at least 48 h. Cells were fixed with 4% formaldehyde, permeabilized with 0.5% Triton X-100, and incubated with an anti-DAPK3 antibody overnight followed by Alexa Fluor 488 secondary antibody. Cells were counterstained with DAPI to label nuclei. The presence of DAPK3 was visualized under an immunofluorescence microscope [20], [21], [22], [23].

#### Xenograft studies

All animals were housed under pathogen-free conditions, and experiments were performed in accordance with Institutional Animal Care and Use Committee approval, Texas tech university health sciences center, El Paso, Texas. Balb/c athymic nude mice (nu/nu) were purchased from the Jackson Laboratory and used at 6–8 weeks of age. For tumor xenograft studies, pCMV/C4-2B or AKT/C4-2B cells (1.5~2.5 × 10^6^) in a 50-µl final volume matrigel matrix were injected subcutaneously into separate flanks of the mouse (6–8 animals). The mice were monitored twice weekly, and tumor volumes were measured once a week.

### Prostate tissue specimens

A total of 29 formalin fixed paraffin embedded (FFPE) tissues with matching controls, and BPH (11 cases) with clinical diagnoses were selected for this study. Prostate specimens were collected from the Department of Pathology under the approval of Committee for the Protection of Human Subjects in Research and Institutional Review Board at University of Louisville, and the experiments were carried out in accordance with the approved guidelines. The BPH and prostate cancer slides were stained with primary antibodies for pAKT (ser473) and DAPK3 followed by secondary antibody incubation. Specimens were analyzed under a light microscope. The slides were viewed and scored by a board-certified pathologist.

Each tissue core was evaluated for pAKT and DAPK3 staining as follows: Location of staining as either cytoplasmic or nuclear, the pattern of staining was either granular or diffuse, and staining intensity was weak, moderate or strong. Each tissue core was scored on a scale of 0 to 3+ with pAKT and DAPK3 expression in normal tissue defining the baseline score of 1+. A score of 0 was no staining, a score of 1+ was either granular staining or weak staining, a score of 2+ was moderate diffuse staining, and a score of 3+ was strong diffuse staining. A composite score for each tissue core was calculated by multiplying the two values together to normalize the heterogeneity of staining and multifocal nature of the tumor by board certified pathologist.

### Statistical analysis

Statistical analysis was performed with the GraphPad Prism 5.0 (GraphPad Software, Inc., La Jolla, CA, USA). Differences between treatment groups were analyzed using a two-sample Student's t-test for normally distributed variables. P values below 0.05 were considered significant. All differences denoted by asterisks were statistically significant and were encoded in the figures (#: not significant, *P < 0.05, **P < 0.01 and ***P < 0.001). The data were presented as the mean + sd for a minimum of three independent experiments unless stated otherwise.

## Results

### Inverse correlation of AKT activation and DAPK3 expression in human prostate cancer cell lines

**Fig. 1:**
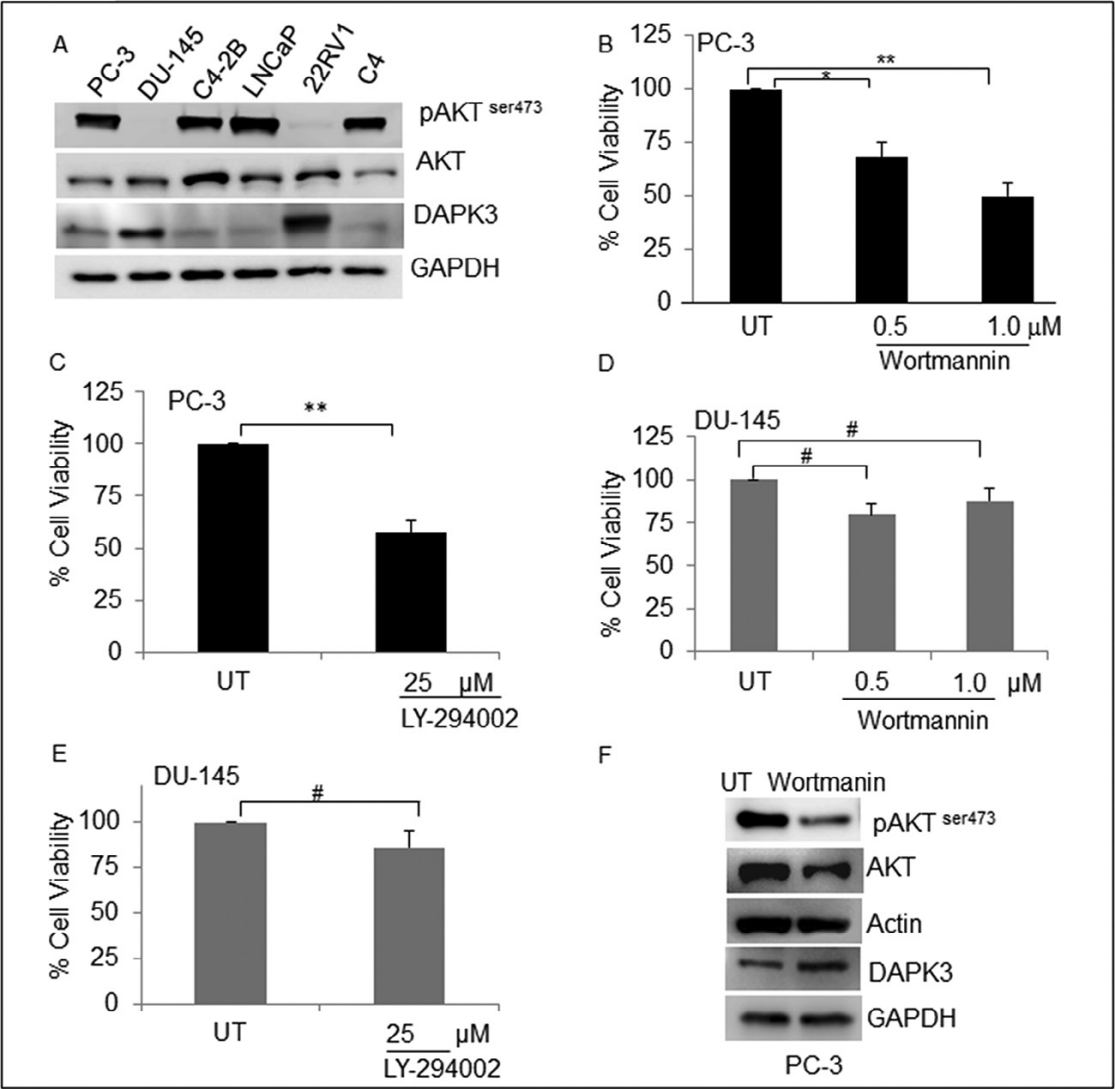
Inhibition of AKT suppresses the CaP growth: (A) to access the basal expression of pAKT and DAPK3, cell lysates were extracted from a panel of CaP cell lines (PC-3, DU-145, C4-2B, LNCaP, 22Rv1 and C4) and subjected to western blot analysis. Different concentrations of wortmannin (B, D) or LY 294002 (C, E) were used for PC-3 and DU-145 cells and subjected to MTT assays. (F) PC-3 cells were treated with wortmannin and western blot analysis was performed for pAKT, AKT, and DAPK3; actin and GAPDH were used as loading controls.

To understand the biological function of DAPK3 in CaP, we first determined the basal expression of DAPK3 in a panel of CaP cell lines by western blot analysis. The DU-145 and CWR22RV1 cells showed higher expression of DAPK3 and low pAKT expression. On the contrary, higher expression of pAKT correlated with lower levels of DAPK3 in PC-3, LNCaP, C4 and C4-2B cells demonstrated(ed) an inverse correlation between pAKT and DAPK3 expression in CaP cell lines (Fig. 1A).

We next analyzed whether AKT is involved in the regulation of DAPK3 expression; we selected PC-3 (PTEN^−^) and DU-145 cells (PTEN^+^). These cell lines were treated with PI3K inhibitors either wortmannin or LY-290042, and cell viability was measured. In PC-3 cells, both wortmannin and LY significantly inhibited the viability of PC-3 cells (Fig. 1B and C). As expected, both PI3K inhibitors fail to alter the cell viability significantly in DU-145 cells (Fig. 1D and E). To confirm whether the inhibition of AKT facilitates DAPK-3 induction in PC-3 cells, we performed western blot analysis. Inhibition of pAKT and induction of DAPK3 were apparent in PC-3 cells. This suggests a possible negative regulation of DAPK3 function by AKT (Fig. 1F).

### AKT negatively regulates DAPK3 expression

**Fig. 2:**
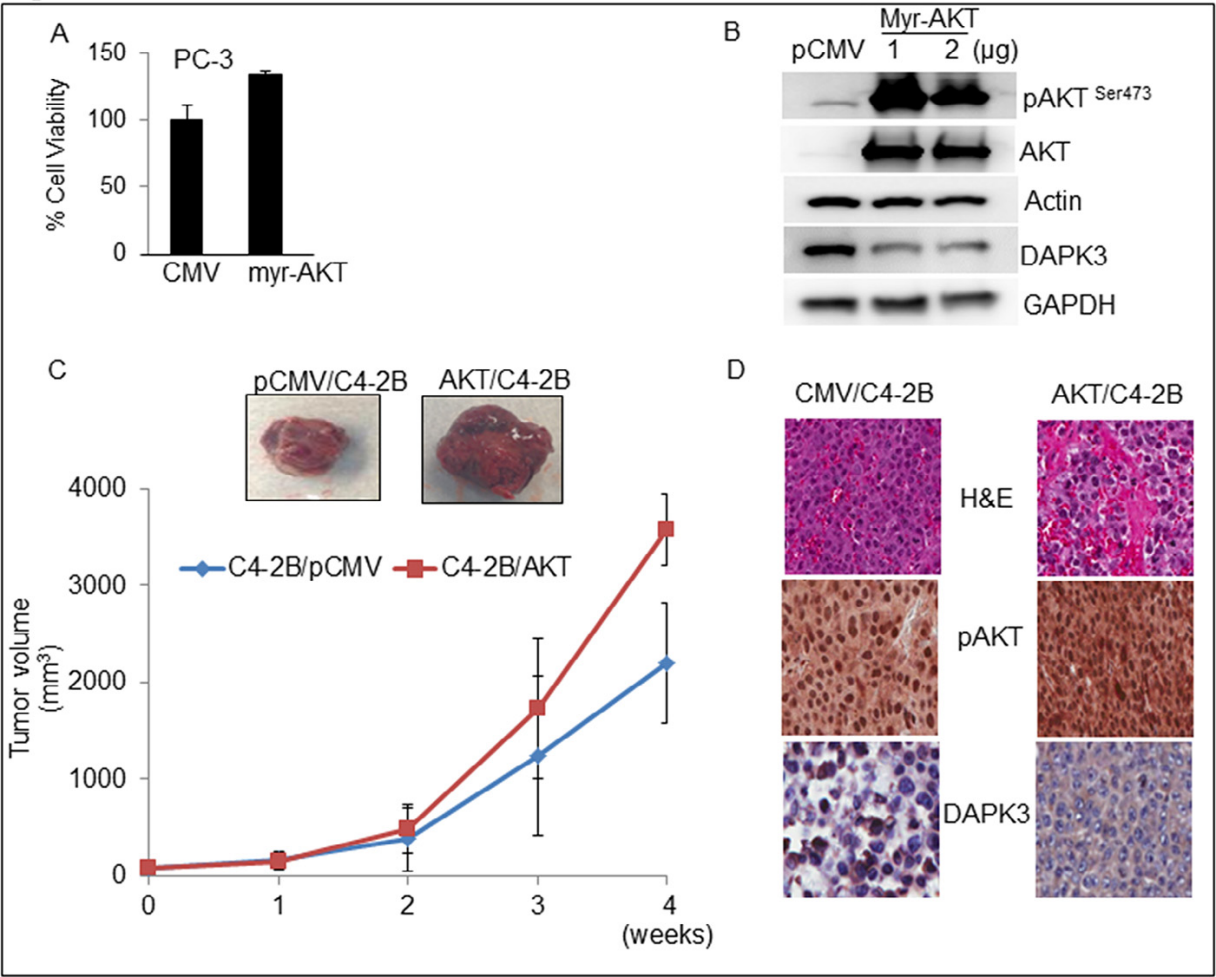
AKT negatively regulates DAPK3 in CaP cells. (A) PC-3 cells were transiently transfected with myr-AKT or empty vector (CMV) and subjected to MTT assay. (B) PC-3 cells were transiently transfected with myr-AKT and empty vector (CMV), and cell lysates were prepared for western blot analysis for pAKT, AKT, and DAPK3 proteins. *β-actin* or GAPDH was used as a loading control. (C) For xenograft studies, C4-2B/pCMV or C4-2B/AKT cells in a final volume (1.5 × 10^6^/50 µl ) were injected subcutaneously in the flanks of mice. The mice were monitored twice weekly, and tumor volumes were measured once a week for 4 weeks. A line graph represents the tumor growth and volume (mm^3^) of C4-2B/pCMV and C4-2B/AKT tumors. (D) Xenograft tumors were analyzed for H&E as well as IHC for pAKT and DAPK3.

To confirm whether overexpression of AKT can alter the expression of DAPK3, we transiently overexpressed AKT in PC-3 cells and performed cell viability assay. An increased proliferation (35%) was observed in AKT/PC-3 as compared to CMV/PC-3 cells (Fig. 2A). We next confirmed overexpression of AKT, which downregulated DAPK3 expression in PC-3 cells by western blot analysis (Fig. 2B). To confirm these findings with in vivo models, we stably overexpressed AKT in C4-2B cells for xenograft studies. CMV or AKT stably over expressing C4-2B cells were implanted subcutaneously into the flank of nude mice. The tumors were allowed to grow, and tumor growth was monitored once a week. As expected, AKT-overexpressing tumors showed 2–3-fold faster growth than pCMV-expressing C4-2B tumors (Fig. 2C). Nuclear localization and a higher expression of pAKT and low levels of DAPK-3 were seen in AKT-overexpressing tumors as compared to control tumors (Fig. 2D). The in vitro and in vivo results confirm that activated AKT downregulated DAPK3 expression and its function in CaP.

### Overexpression of DAPK3 induces pro-apoptotic function

**Fig. 3:**
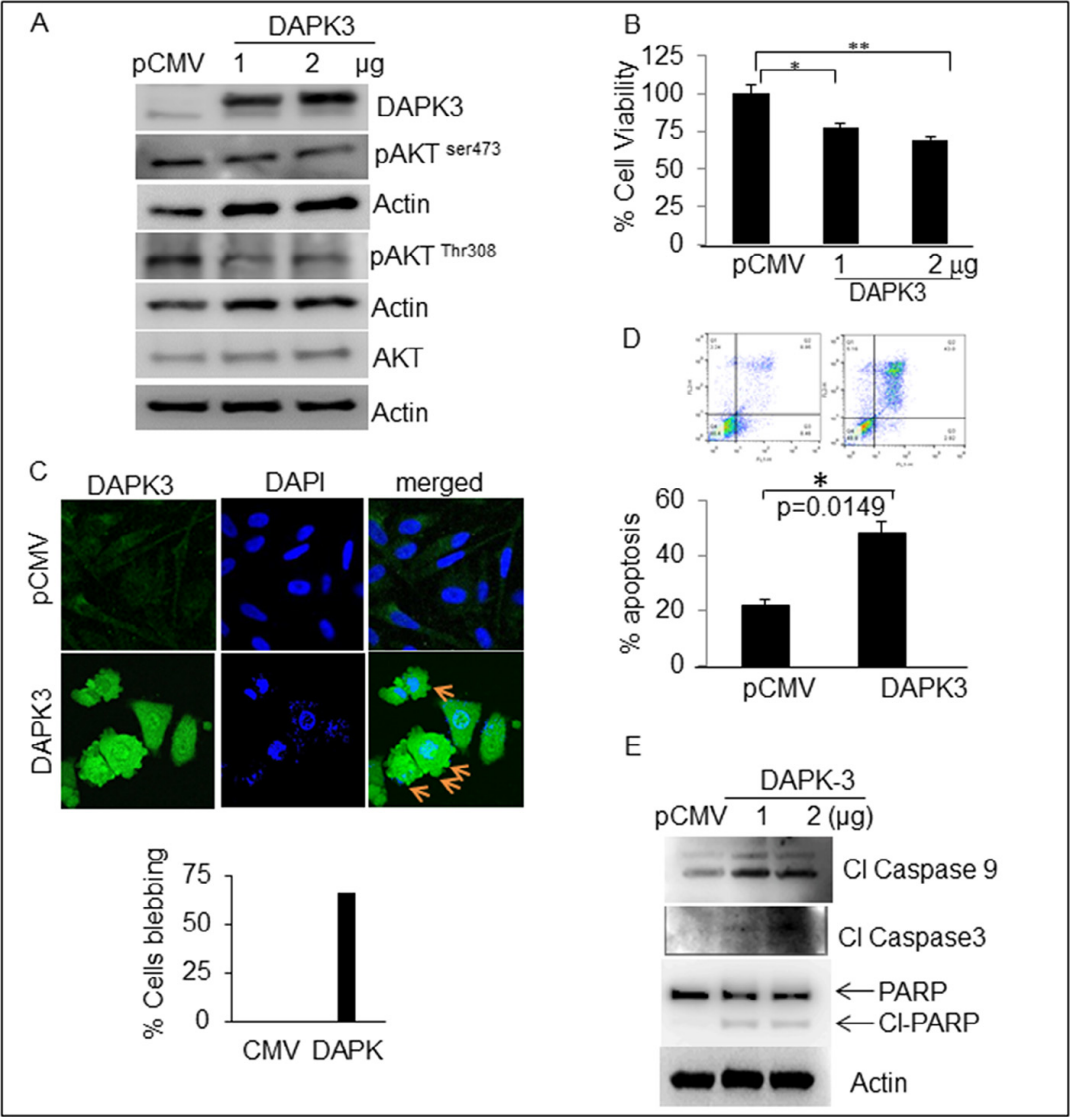
Over-expression of DAPK3 induces apoptosis in CaP cells. (A) PC-3 cells were transiently transfected with DAPK3 and empty vector for 48 h. The cell lysates were subjected to western blot analysis for DAPK3 and pAKT expression. β-Actin or GAPDH was used as a loading control. (B) To check cell proliferation, transfected PC-3 cells with both CMV and DAPK3 were plated in 24-well plates and allowed to grow for 24 h followed by an MTT assay. (C) DAPK3 over-expression causes membrane blebbing in PC-3 cells as detected by confocal microscopy and (D) annexin V-FITC staining. (E) PC-3 cells were transiently transfected with DAPK3 and empty vector for 48 h. The cell lysates were subjected to western blot analysis for cleaved caspase-3, -9 and PARP. β-Actin was used as a loading control.

To confirm the pro-apoptotic function of DAPK3, we ectopically over-expressed DAPK3 in PC-3 cells. The MTT analysis suggests inhibition of cell viability in DAPK3 over-expressed cells in a dose-dependent manner (Fig. 3B). Western blot analysis confirmed the over-expression of DAPK3 and concomitant down-regulation of pAKT (Ser 473 & Thr 308) expression in PC-3 cells (Fig. 3A). Next, we analyzed cell morphology of DAPK3 over-expressing cells by confocal analysis. Interestingly, DAPK3 over-expressing cells exhibit apoptotic-like morphology including cell shrinkage, plasma membrane blebbing and condensation in nuclear chromatin. These are hallmarks of programmed cell death (Fig. 3C). These results were further confirmed by annexin V-FITC staining (Fig. 3D).

Next, we analyzed pro-apoptotic proteins to confirm whether DAPK3 over-expressed cells exhibit classical apoptotic pathways. Our results showed increased apoptosis, which correspond to caspase-9 and 3 and PARP cleavage. This suggests that activation of DAPK3 induces cell death in CaP cells (Fig. 3E). Finally, we silenced DAPK3 by siRNA and determined whether inhibition of DAPK3 reverses pAKT expression in cancer cells. We observed that silencing of DAPK3 restored the AKT activation in CaP cells suggesting a possible feedback loop of activation between these two kinases. This may decide the fate of the cells (data not shown).

### Inverse correlation between pAKT and DAPK3 expression in human CaP tissues

**Fig. 4:**
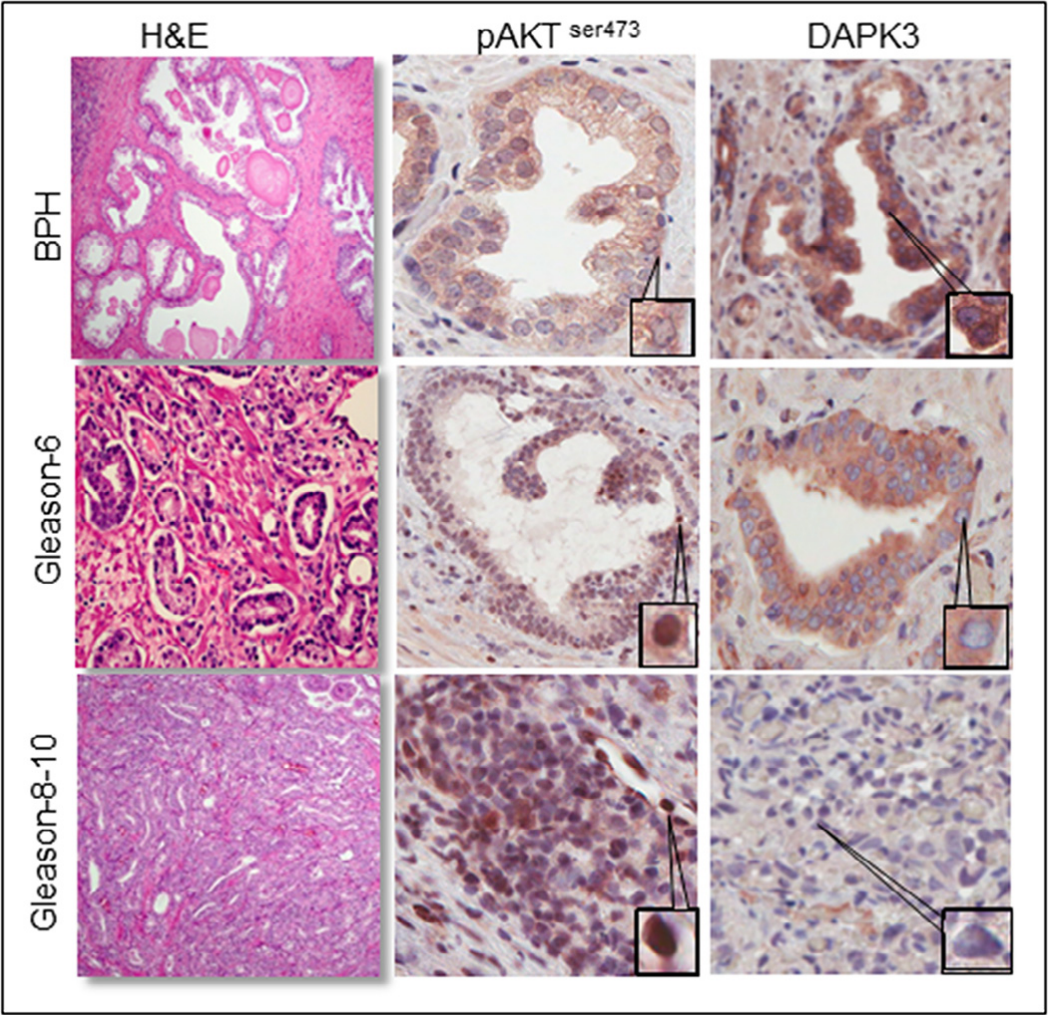
Human CaP tissues illustrating the expression of pAKT and DAPK3 (H&E staining for human CaP; IHC for pAKT and DAPK3 in representative sections of human CaP).

In human CaP tissues, a gradual increased expression of pAKT from BPH to higher Gleason stages is seen concomitantly with a steady decrease in DAPK3 expression. More precisely, pAKT expression was low in BPH, whereas DAPK3 was strongly expressed in cytoplasm; however, in carcinoma, we noticed a higher expression for pAKT in the nucleus in higher Gleason score and decreased expression of DAPK3 in higher Gleason score samples (Fig. 4).

## Discussion

We demonstrated an inverse correlation between AKT activation and DAPK3 function in preclinical and human tumor tissues. Inhibition of AKT or overexpression of DAPK3 inhibits the growth of CaP cells and confirms that AKT could be a negative regulator of DAPK3 in CaP.

Activated AKT plays a critical role in inhibiting apoptosis and concomitantly promotes pro-survival function in many epithelial cancer types including CaP [24], [25], [26]. We and others have reported that AKT expression was correlated with CaP progression. It may also influence the transition to CRPC [27], [28]. Hence, AKT is an attractive therapeutic target for CaP. Many small molecule inhibitors against AKT activation have been developed, and promising results were achieved in preclinical [29], [30] and clinical trials [31]. Activated AKT also regulates many pro-apoptotic factors and phosphorylates FOXO3a at Thr^24^, Ser^256,^ or Ser^318^ residues. This in turn inhibits pro-apoptotic activity such as cell cycle arrest by downregulating p27, GADD45 and BIM [8], [32], [33]. Recently, we demonstrated that AKT negatively regulates the pro-apoptotic function of prostate apoptosis response-4 (Par-4) by inactivating FOXO3a. Similarly, in this study, DAPK3 was shown to be another tumor suppressor that may be inhibited by AKT.

Inhibition of AKT by wortmannin restores the pro-apoptotic function of DAPK3 that resulted in growth inhibition in CaP cells. The inverse correlation of AKT and DAPK3 may suggest that DAPK3 could be a substrate for AKT, which resulted in inhibition of DAPK3 function. Interestingly, silencing DAPK3 reverted the pAKT expression in PC-3 cells and suggests there might be a feedback loop mechanism; these two kinases may regulate each other's function. More studies are needed to determine how these kinases interact and decide the fate of the cell in cancer.

Increased expression of pAKT can be clinically correlated to the severity and prognosis of the patient's CaP. In our studies, a higher expression of pAKT was seen in Gleason 6–10 CaP versus the BPH – this confirms the oncogenic role of AKT in CaP. In the Gleason 8–10 patients, the increased expression of pAKT was seen in patients with poor prognosis including bone metastasis and pathologic spine metastasis, which required palliative radiation treatment. Patients undergoing hormone deprivation with radical orchiectomy, LHRH agonist, or treatment with anti-androgen agents (bicalutamide) with associated metastatic CaP also have elevated AKT in the nucleus of biopsied tissues. Patients with aggressive forms of CaP may be more likely to have hormone refractory tumors and elevation in AKT may be the driving force of disease progression. Therefore, the implication for castrate-resistant CaP needs further analysis, but might have similar behavior.

DAPK3 induces apoptosis either dependent or independent of caspase signaling. Mutation and deletion of DAPK3 is a major setback that can lead to a survival advantage in cancer cells [19]. DAPK3 is a Ca^2+^/calmodulin-regulated serine/threonine kinase that is predominately activated by oxidative stress to enhance cell death via apoptosis [34]. A recent study demonstrated that activation of DAPK3 causes genome instability independent of p53 status in cancer cells [35]. These results may support our finding that over-expression of DAPK3 in p53/PTEN null PC-3 cells causes growth inhibition independent of p53 status. DAPK3 may induce pro-apoptotic function, which highlights its importance as a therapeutic target for CaP. Over-expression of DAPK3 caused extensive membrane blebbing, which is a classical phenomenon of programmed cell death (PCD). It has been reported that ROCK-I activation causes myosin phosphorylation, which in turn constructs the actin cytoskeletons. This leads to plasma membrane blebbing [36]. Similar studies were reported for quinazolinone Schiff base derivatives that induced both intrinsic and extrinsic apoptosis pathways to yield membrane blebbing in MCF-7 cells [37].

On the other hand, the mutation or deletion of DAPK3 accelerates tumor progression in many cancer types. For example, cells harboring mutated DAPK3 may get chemosensitized with ectopic over-expression of wild type DAPK3 in lung cancer models [19]. These results further confirm the tumor suppressor role of DAPK3 in cancer types. Few studies have pointed out that MLC2, histone H3, and Par-4 may act as a substrate in DAPK3 activation. This causes apoptosis in cancer cells [12], [19].

In clinical studies, we found that DAPK3 expression is higher in hyperplasia and gradually decreased expression in high Gleason score sample. Vice versa, pAKT expression was seen in prostate tumor samples. Similar observations were made in gastric carcinoma specimens. We believe more specimens may require to understand the role of DAPK-3 in prostate cancer cells, however, it is evident that inhibition of DAPK3 is higher in higher Gleason-score samples in our results. It is critical that whether low expression of DAPK-3 in higher Gleason could be due to deletion and mutation of DAPK-3 expression in human prostate cancer specimens. Down-regulation of DAPK3 was seen in 111 of 162 gastric cancer cases, and this correlated with invasion, metastasis and poorer prognosis [38]. Hunter's group [17] demonstrated that DAXX transcription factor represses the function of both DAPK1 and DAPK3 in CaP supporting our findings that targeting DAPK3 may be a viable option for CaP treatment. Mutation and deletion of tumor suppressor genes such as p53, PTEN, and Par-4 are seen in CaP. DAPK3 is also a potent tumor suppressor, and its functions are compromised during the progression of CaP. It is possible that DAPK3 could be regulated by many signaling pathways, and reactivating DAPK3 function may prevent and treat CaP.

## Funding

This work was supported by the NIH/NCI R01CA140605, and R01CA138797 (to C. D.).

## Conflict of interest

The authors declare that they have no conflict of interest.
